# Peri-implant soft tissue response to different abutment materials: A randomised controlled clinical trial

**DOI:** 10.6026/973206300221170

**Published:** 2026-02-28

**Authors:** Sarvesh Ingole, Sukhada Pawar, Sowjanya Gunukula, Akshay J Melath, Zameer Pasha, Nishant Visvas Dumont, Krutika Bidkar

**Affiliations:** 1 Department of Dentistry, Bharati Vidyapeeth Dental College, Pune, Maharashtra, India; 2 Department of Dentistry, Founder and Lead Dentist, Swami Dental Care, Pune, Maharashtra, India; 3 Department of Dentistry, Independent researcher, Flomo Dental, 295 Ukiah St, Lewisville TX 75056, USA; 4 Department of Periodontology, Guru Gobind Singh college of Dental Science and Research Centre, Burhanpur, Madhya Pradesh, India; 5 Department of Oral and Maxillofacial Surgery, Consultant in Oral and Maxillofacial Surgery, Durrat Al-Alammi Dental Clinic, Al-Majmaah,Riyadh, Saudi Arabia; 6 Department of Oral & Maxillofacial Surgery, Mahatma Gandhi Post Graduate Institute of Dental Sciences, Pondicherry University, Pondicherry, Pondicherry, India; 7 Department of Periodontology, Aditya dental college, Beed, Maharashtra, India

**Keywords:** Bleeding on probing (BoP), Peri-implant soft tissue, transmucosal abutment materials, titanium, zirconia

## Abstract

Peri-implant soft tissue response varies with different transmucosal abutment materials, but their comparative effects on clinical, histologic and microbiologic markers are not well understood. This randomized, controlled, split-mouth clinical trial compared titanium (Ti), zirconia (Zr) and titanium with a TiO_2_ bioactive coating (Ti+TiO_2_) in 50 systemically healthy participants. Over 12 months, zirconia and Ti+TiO_2_ showed lower bleeding on probing (BoP) and reduced inflammatory markers compared to titanium, with Ti+TiO_2_ demonstrating denser connective tissue attachment. Zirconia was preferred for esthetics. This study advances knowledge by highlighting the comparative advantages of Ti+TiO_2_ and zirconia in peri-implant tissue response.

## Background:

A stable peri-implant soft tissue seal is crucial for the long-term success of dental implants, as it plays a key role in preventing microbial ingress, peri-implant bone loss and subsequent implant failure [1]. Over the years, titanium abutments have been the standard material used for implant restoration due to their strength, biocompatibility and ability to integrate with bone. However, concerns related to mucosal discoloration and the esthetic outcome, particularly in the anterior zone, have led to increased interest in alternative materials [2]. Zirconia abutments, in particular, have emerged as a promising option due to their excellent color match to natural gingiva, which can be particularly advantageous in patients with thin gingival biotypes where esthetics are a major concern [3]. Additionally, surface-modified titanium abutments, such as those coated with titanium dioxide (TiO_2_), have been developed with the aim of improving soft tissue integration, enhancing the epithelial/connective tissue attachment and potentially reducing inflammation around the implant site [4]. Clinical and spectrophotometric studies suggest that zirconia offers a superior color match to natural gingiva compared to titanium, which can significantly improve pink esthetic scores (PES) in specific cases. These advantages are particularly evident in patients with thin gingival biotypes, where the translucency and natural color of zirconia better mimic the surrounding tissues [5]. Despite these potential benefits, systematic reviews and randomized controlled trials (RCTs) have shown only modest or inconsistent differences between zirconia and titanium abutments when measured by common clinical indices such as probing depth, bleeding on probing (BoP) and marginal bone levels [6]. These mixed findings suggest that while zirconia may offer some esthetic advantages, it may not consistently outperform titanium in terms of clinical parameters. This highlights the necessity for well-designed RCTs that incorporate clinical, molecular and histologic endpoints to provide a comprehensive assessment of the soft tissue response to different abutment materials [7].

Furthermore, recent advancements in surface engineering, particularly the use of TiO_2_ nanoporous or sol-gel coatings and anodization, have shown promise in enhancing epithelial and connective tissue attachment to titanium [8]. These surface modifications may also reduce pro-inflammatory signaling, contributing to better soft tissue health and a reduction in inflammation around the implant. However, clinical data supporting the efficacy of these modified titanium abutments remain limited and more research is needed to validate their potential benefits [9]. Experimental split-mouth trials, where multiple materials are compared within the same subject, offer a valuable advantage by providing high internal control for patient- level confounders. This approach allows for more accurate comparisons and also permits histologic sampling in a controlled setting, which can provide detailed insights into the tissue-level responses to different materials. Given the need for further evidence on the clinical, molecular and histologic effects of different abutment materials, this trial was designed to test the hypothesis that zirconia and TiO _2_-coated titanium abutments produce equal or improved peri-implant soft tissue health compared with standard titanium abutments [10]. Therefore, it is of interest to determine the comparative effectiveness of these materials in improving peri-implant soft tissue health, reducing inflammation and enhancing esthetic outcomes, ultimately contributing to the long- term success and predictability of dental implants.

## Materials and Methods:

This study is a single-center, randomized, controlled, examiner-blinded, split-mouth clinical trial with a 12-month follow-up, designed to evaluate the peri-implant soft tissue response to different abutment materials. Ethical approval was obtained from the relevant institutional review board and participants provided written informed consent. Adults aged 18-75 years, systemically healthy (ASA I-II), non-smokers or smoking ≤5 cigarettes/day and requiring at least two adjacent single implants with healed sites were included in the study. Exclusions were made for individuals with uncontrolled systemic diseases, active periodontitis, heavy smoking (>5/day), pregnancy, recent use of antibiotics, bisphosphonate therapy, or allergies to the materials used in the study. A computer-generated random sequence was used to allocate abutment types to implant sites in a split-mouth design, ensuring that each participant received titanium (Ti), zirconia (Zr) and titanium with TiO_2_-coated titanium abutments. For those requiring three implants, balanced block randomization was employed to ensure an even distribution across groups. The allocation process was concealed in sealed envelopes. Implants were placed following the manufacturer's protocols and after 3 months of osseointegration, the transmucosal abutments were placed according to the following groups: Ti (machined commercially pure titanium), Zr (monolithic zirconia) and Ti+TiO_2_ (titanium with TiO_2_ nanoporous coating). Standardized oral hygiene instructions were given and professional prophylaxis was performed at each visit. Clinical measurements were taken at baseline (abutment placement), 3, 6 and 12 months by a calibrated, blinded examiner. These included plaque index (PI), modified gingival index (mGI), probing depth, bleeding on probing (BoP), mucosal recession, width of keratinized mucosa and Pink Esthetic Score (PES) for anterior cases. In addition to clinical assessments, peri-implant crevicular fluid (PICF) was collected and stored at - 80°C for IL-1β and IL-6 quantification using ELISA. Submucosal plaque samples were collected using paper points and DNA was extracted to perform qPCR analysis for key periopathogens such as *Porphyromonas gingivalis, Tannerella forsythia* and *Treponema denticola.* In a subset of 20 participants, soft tissue punch biopsies were obtained at 3 months for histologic analysis, including inflammation grading and vascular density measurements, to assess tissue responses in a controlled manner. The primary outcome of the study was the change in BoP (%) at 12 months, while secondary outcomes included levels of IL-1β/IL-6, PES, probing depth, histologic inflammation scores and microbial counts. Sample size calculations based on pilot data indicated that 42 patients were needed to detect a 15% absolute difference in BoP with 80% power and an α=0.05. To account for potential dropouts, 50 participants were recruited. Adverse events were monitored throughout the study and biopsies were limited to consenting participants, with all data de- identified to maintain confidentiality. Data analysis was conducted using SPSS. Pre-analysis diagnostics included checks for normality using histograms, Q-Q plots and the Shapiro-Wilk test. Paired t-tests, repeated-measures ANOVA, linear mixed-effects models and non-parametric tests were used to analyze continuous outcomes, while generalized linear mixed models were applied to categorical outcomes. Multiple imputation methods were used for missing data and post hoc sensitivity analyses were performed to explore different analysis approaches. This study's robust analytic plan ensures transparent and accurate assessment of the outcomes in this split-mouth RCT design, allowing for a reliable comparison of the different abutment materials in terms of clinical, histologic and microbiologic markers.

## Results:

In the clinical outcomes, the mean BoP at 12 months was 20.1% (±9.4) for the Ti group, 13.8% (±8.2) for the Zr group and 14.6% (±8.7) for the Ti+TiO_2_ group (Table 1). The analysis showed that both Zr and Ti+TiO_2_ had significantly lower odds of bleeding compared to Ti, with an odds ratio of approximately 0.65 (95% CI 0.42- 0.99, p=0.04). Regarding PES, zirconia had a higher mean score of 7.1, compared to 6.3 for titanium, with a statistically significant difference (p=0.02) (Table 2).In terms of molecular markers, the median reduction of IL-1β at 12 months was -2.4 pg/µL for Ti, -5.1 pg/µL for Zr and-5.7 pg/µL for Ti+TiO_2_ (Table 3). The Ti+TiO_2_ and Zr groups showed a significant reduction in IL-1β compared to Ti (p~0.03). However, the IL-6 differences were smaller and not statistically significant. In histology (subset n=20), the inflammatory cell density was lower in Ti+TiO_2_ specimens (median grade 1) compared to Ti (median grade 2), with a p-value of 0.03 (Table 4). This suggests that Ti+TiO_2_ had less inflammation at the tissue level. The microbial counts for *P. gingivalis* and other red-complex species showed no clinically meaningful differences across the groups at 12 months (Table 5). Regarding adverse events, mild transient mucosal irritation occurred in 6% of participants across all groups, with no serious adverse events reported (Table 6).

## Discussion:

This randomized split-mouth trial evaluated whether zirconia or TiO_2_-coated titanium abutments yield superior peri-implant soft tissue health compared with standard titanium over 12 months, using clinical, molecular, microbiologic and histologic endpoints. In comparison with van Brakel *et al.* (2012) [11], who found no major differences in vascular density and inflammation between zirconia (Zr) and titanium (Ti) in their histologic study, our results showed that Ti+TiO _2_ had significantly lower inflammatory cell density compared to Ti (p=0.03), suggesting a slight advantage for Ti+TiO _2_ in reducing inflammation. However, both studies found comparable vascular density across materials, which indicate consistency in soft tissue response in terms of vascularity. Similarly, Cosgarea *et al.* (2015) [12], demonstrated that zirconia had a better tissue color match than titanium in their spectrophotometric RCT. Our study found a significant difference in Pink Esthetic Score (PES), with zirconia showing a higher score by 0.8 points compared to titanium (p=0.02), driven by improved tissue color and contour match, especially in anterior cases. This reinforces the notion that zirconia offers superior esthetic outcomes in the aesthetic zone (Table 2). In Linkevicius and Vaitelis 2015 [13], a systematic review indicated that zirconia only had limited clinical advantages over titanium for most soft tissue metrics. Our study found that while probing depth differences were not statistically significant, zirconia and Ti+TiO_2_ both exhibited lower bleeding on probing (BoP) compared to Ti, suggesting that zirconia may provide a better clinical outcome in terms of soft tissue health (Table 1). This is consistent with Linkevicius and Vaitelis assertion that the clinical benefits of zirconia are modest but noticeable in some aspects, such as bleeding control. Sanz-Martín *et al.* (2018) [14] highlighted the impact of material and surface treatment on BoP in their meta-analysis.

In our study, Ti+TiO_2_ exhibited a significant reduction in IL-1β levels and a lower BoP compared to Ti (p~0.03), which supports Sanz-Martín *et al.*'s conclusion that surface modifications, like TiO_2_ coatings, are influential in improving soft tissue outcomes (Table 3). This suggests that surface treatments such as TiO _2_ can enhance soft tissue healing and reduce inflammation. Finally, comparing our findings with Enkling *et al.* (2022) [15], who reviewed TiO_2_ coatings enhancing soft tissue healing, our study corroborated this by demonstrating that Ti+TiO_2_ resulted in reduced inflammatory cell density and lower IL-1β levels, further supporting the role of TiO_2_ coatings in improving peri-implant tissue health. This confirms the growing body of evidence regarding the benefits of TiO_2_ coatings in enhancing soft tissue integration. Together, these studies present a nuanced picture: while zirconia tends to offer aesthetic benefits and certain microcirculatory advantages and TiO_2_-modified titanium may favorably influence soft tissue attachment and early inflammatory signaling, gross clinical endpoints (PPD, recession, survival) are often similar within short-term follow-up. Our trial's molecular and histologic signals support biologic plausibility for modest early advantages of Ti+TiO_2_ and Zr which might translate into clinically meaningful differences over longer durations or in high-risk patients.

## Conclusion:

In this 12-month randomized split-mouth trial, zirconia and TiO_2_-coated titanium abutments showed modest benefits over standard titanium in peri-implant esthetics and inflammation (lower BoP and IL-1βbeta;). Core clinical parameters like probing depth, mucosal recession and implant survival remained similar. These early biologic and histologic findings warrant further long-term studies to assess their impact on peri-implant disease prevention and esthetic outcomes.

## Figures and Tables

**Table 1:** Bleeding on Probing (BoP) at 12 months

**Group**	**Mean BoP (%)**	**Standard Deviation**
Ti	20.1	±9.4
Zr	13.8	±8.2
Ti+TiO_2_	14.6	±8.7

**Table 2:** Pink Esthetic Score (PES) Comparison

**Group**	**Mean PES**	**p-value**
Ti	6.3	
Zr	7.1	0.02

**Table 3:** IL-1β Reduction in Peri-implant Crevicular Fluid (PICF) at 12 Months

**Group**	**Median IL-1β Reduction (pg/µL)**
Ti	-2.4
Zr	-5.1
Ti+TiO_2_	-5.7

**Table 4:** Histology Inflammatory Cell Density at 3 Months (Subset n=20)

**Group**	**Median Inflammatory Cell Density (Grade)**	**p-value**
Ti	2	
Zr	1	
Ti+TiO_2_	1	0.03

**Table 5:** Microbial Counts for *P. gingivalis*at 12 Months

**Group**	***P. gingivalis*Count (copies)**
Ti	No significant difference
Zr	No significant difference
Ti+TiO_2_	No significant difference

**Table 6:** Adverse Events

**Adverse Event**	**Ti (%)**	Zr (%)	Ti+TiO_2_(%)
Mild transient mucosal irritation	**6%**	**6%**	**6%**
